# The rheology of a growing leaf: stress-induced changes in the mechanical properties of leaves

**DOI:** 10.1093/jxb/erw316

**Published:** 2016-09-20

**Authors:** Michal Sahaf, Eran Sharon

**Affiliations:** The Racah Institute of Physics, The Hebrew University of Jerusalem, Jerusalem, Israel

**Keywords:** Growth, leaf, morphogenesis, mechanical stress, remodelling, rheology.

## Abstract

We study *in situ* the mechanics and growth of a leaf. Young *Nicotiana tabacum* leaves respond to applied mechanical stress by altering both their mechanical properties and the characteristics of their growth. We observed two opposite behaviours, each with its own typical magnitude and timescale. On timescales of the order of minutes, the leaf deforms in response to applied tensile stress. During this phase we found a high correlation between the applied stress field and the local strain field throughout the leaf surface. For times over 12 hours the mechanical properties of the leaf become anisotropic, making it more resilient to deformation and restoring a nearly isotropic growth field despite the highly anisotropic load. These observations suggest that remodelling of the tissue allows the leaf to respond to mechanical perturbations by changing its properties. We discuss the relevance of the observed behaviour to the growth regulation that leads to proper leaf shape during growth.

## Highlight

A growing leaf is described as a viscoelastic material with properties that change in response to mechanical stress, leading to changes in the magnitude and anisotropy of the growth field.

## Introduction

Leaf growth is a complex process, which involves many biological and chemical cycles leading to the final shape of the leaf. From a mechanical point of view, the growing leaf can be viewed as a sheet of active material with effective mechanical properties that are determined by the underlying biological activity. Such an effective rheology should strongly affect the leaf’s growth process and its regulation. Indeed, when studying morphogenesis, the mechanical aspect cannot be ignored. It is now well known that, in many cases, the final shape and properties of an organ, or even an entire organism, are greatly influenced by mechanics (Gjorevski and Nelson, 2010; Mirabet *et al.*, 2011; Kierzkowski *et al.*, 2012; Wolff, 2012; Bozorg *et al.*, 2014; Shawky and Davidson, 2015). However, the spatiotemporal characteristics of the mechanical response are not known. In particular, it is not clear what characteristics of the effective rheology lead to the robustness of leaf growth. Such characteristics cannot be measured on a single-cell level and require *in vivo* study at the tissue level.

In growing leaves, cell division ceases almost completely at an early stage (Poethig and Sussex, 1985), and most of the lateral growth, by an order of magnitude and more, is achieved by cell expansion. Growth at this stage is described (Alberts *et al.*, 2002; Cosgrove, 2005) as a metabolically controlled yielding of the cell wall to the osmotic turgor pressure; specific proteins are responsible for loosening the cell wall so that it may be extended, and new material is integrated into the wall, thus maintaining its thickness and strength.

The cell wall is a complex structure. Even so, mechanically it is commonly described (Cosgrove, 1993) as a thin shell composed of a viscoelastic material. Because it is the main contributor to the mechanical strength of the cell, it would make sense to expect viscoelastic behaviour from the intact leaf tissue. However, effects such as poroelasticity, as well as possible biological active processes, play an important role in the mechanical behaviour of the leaf (Dumais and Forterre, 2012). One can ask whether the integrated effect of such biological and chemical processes may be described by state-dependent viscoelastic parameters of the whole tissue, which may differ from those of the cell wall material itself. In this work, we take the first steps towards determining the large-scale rheology of a growing leaf and its possible relation to growth patterns.

From a mechanical point of view, leaves may be approximated as two-dimensional sheets. Therefore, their growth and stress accumulation differ from those of roots and hypocotyls, which are nearly one-dimensional organs. One-dimensional objects that grow non-uniformly are free to deform according to the growth distribution and therefore do not develop internal stresses. Contrarily, non-uniform growth of two-dimensional sheets can be incompatible, leading to the accumulation of stresses within the sheet (Efrati *et al.*, 2009*a*). In some cases the accumulation of stress serves as a way to generate large-scale motion of the plant organs (Forterre *et al.*, 2005; Elbaum *et al.*, 2007; Armon *et al.*, 2011); however, excess or irregular stress in leaves can lead to buckling, causing undesired deformations (Efrati *et al.*, 2009*b*; Sharon and Efrati, 2010).

Leaf growth occurs simultaneously in multiple cells. This process is far from smooth (Elsner *et al.*, 2012), and is therefore bound to create local stresses, due to the differences in the growth rates of neighbouring cells. Such stresses, if not relieved, will cause random buckling throughout the area of the leaf. In a healthy plant this is not the case, and the highly irregular growth pattern is balanced out to produce a well-defined leaf shape. Certain pathogens (Cohen, 1995), as well as genetic manipulations (Nath *et al.*, 2003; Palatnik *et al.*, 2003), may lead to random changes in the shape of the leaf, including its buckling into three-dimensional configurations, indicating that one or more shape-regulating mechanisms are perturbed. However, little is known about the nature of such mechanisms.

Various works (Jaffe, 1980; Coutand *et al.*, 2000; Hellgren *et al.*, 2004; Braam, 2005) have shown a wide range of mechanically induced effects in plants. In particular, it has been shown (Nakayama *et al.*, 2012) that auxin transport is affected by mechanical stimuli. Given that plants respond to some mechanical stimuli, it is plausible that mechanisms which serve to conserve the leaf’s shape may be initiated by the stress (or strain) that results from spatial differences in the growth rate. Such a response will manifest as a dependence of growth rate or isotropy on the local stress. This mechanism can be either passive, similar to the response of a viscoelastic material to stress, or active, involving various biological functions that modify the properties of the leaf (Sampathkumar *et al.*, 2014). A combination of these two is most likely.

Recently it has been shown (Uyttewaal *et al.*, 2012) that the orientation of cortical microtubules is related to naturally occurring mechanical stresses resulting from the curvature of the meristem. Cortical microtubules are considered (Paredez *et al.*, 2006) to have an effect on the orientation of cellulose microfibrils in the cell wall. Such changes in the structure of the wall may lead to changes in the mechanical properties and growth pattern of the leaf (Gibson and Gibson, 2012).

In this work we directly study the effect of mechanical loading on leaf growth and the mechanical properties of the leaf tissue, and consider its possible implications to leaf morphogenesis. Using high-resolution measurements of the local growth of the leaf, while applying a controlled external load, we analyse the correlations between the strain and stress fields and measure the rheology of a growing leaf. Our measurements show that the leaf is not a passive material, but an active, responsive one, which remodels itself according to the mechanical stimulation it is exposed to during its growth process.

## Materials and methods

Deducing the local stress state in a naturally growing leaf from local strain measurements is nearly impossible without damaging the leaf (i.e. inserting cuts and holes). Inducing a dominant external, well-controlled mechanical stress provides a more practical way to observe the effect of stress on growth rate and direction. Here we apply tensile stress to one side of a growing leaf of wild-type tobacco (*Nicotiana tabacum*) and study the growth of the loaded area compared to the other side of the leaf, which serves as a control, and compared to its previous loose state. All measurements described in this work were performed on young, first to third leaves (cotyledons excluded), with a length of 1.5–3cm, while still intact and attached to the plant. Plants were grown in soil (peat, tuff and coconut fibres) under short-day conditions and then moved to long-day conditions for measurement.

**Fig. 1.**
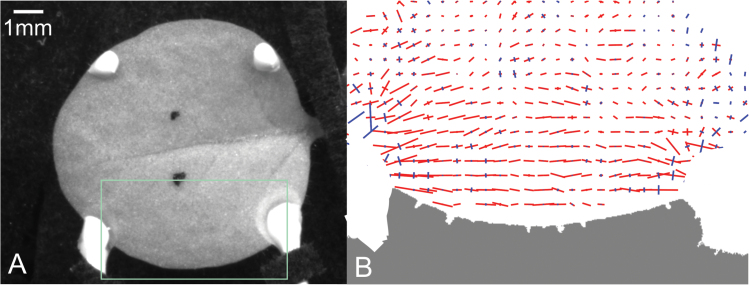
**(A)** A young tobacco leaf attached to the stretching tubules. Bright and darks spots define the ROI, one that is subjected to stress (at the lower part of the picture) and one that serves as a control (upper part of the picture). **(B)** An example of the growth tensor in the stretched area over the first 20 minutes after stress is applied. Elongation is indicated in red, contraction in blue.

We applied controlled tensile force using two calibrated elastic glass tubules (see Fig. 1A). The tubules were attached to the leaf edge by two-part epoxy glue, with their other end mounted on motorized stages. A known displacement was applied by the stage to the end of each tubule, pulling them apart, while the movement of their other ends, attached to the leaf, was monitored by a camera (with spatial resolution of ~10 µm). In this way, both the applied force and the resulting strain of the leaf could be measured and controlled in real time (see Supplementary data: Figures S1 and S2).

Various temporal force profiles could be applied, such as constant force (creep experiment), or sinusoidal force required for dynamic mechanical analysis.

Two cameras were used to take images of the surface of the leaf. The images were then processed using a particle image velocimetry (PIV) technique, which tracks changes between images and provides the displacement field of elements of the leaf surface. The natural texture of the leaf surface serves as the small scale field, which is required by the algorithm. In addition to the horizontal displacement field, we obtained the topography of the leaf surface at each measurement time. The measurement was performed by a stereoscopic analysis: The two cameras were separated by 9cm and oriented with a 10° angle difference, obtaining simultaneous images of the leaf at Δt time intervals. Every two simultaneous images were compared, and the apparent local horizontal displacement between them (stemming from the different orientation of each camera) determined the vertical coordinate of the surface via a stereoscopic analysis, thus completing the three-dimensional surface map z(x,y) . PIV analysis of sequential three-dimensional leaf surfaces provided the three-dimensional local displacement field from which the strain tensor field was obtained (see Fig. 1B for an example of a growth field, and Supplementary data: Figures S3 and S4 for examples of three-dimensional and growth calculations). This field included both naturally occurring growth and stress-induced deformation. The applied stress field (not normalized by the leaf cross section) was calculated numerically using the finite element method (MATLAB) to provide local, as well as global, stress-strain relations. The exact mechanical stress (force over area) depends on the distribution of load-bearing elements in the leaf, which are mostly the epidermis and the vascular system (Onoda *et al.*, 2015). Due to the complexity of the tissue’s cross-section and in order to avoid inaccuracies, we refer in our results to the applied force rather than the stress.

Images were taken during day time only, so as to keep the night time dark (IR illumination of 850nm was used to monitor the force), and so our data consist of measurements performed for several hours after stretching, and then again during the next day. Information on naturally occurring changes during these two periods could be extracted from the control side, so that all final results presented in this work were normalized by the average growth rate of the control side.

Dynamic mechanical analysis (DMA) was used to measure the mechanical properties of the leaf. In this mode of operation, we applied a periodic stress with a sinusoidal profile of frequency ω and amplitude $\sigma_{0}.$ The leaf responded by straining with amplitude $\varepsilon_{0},$ but due to the viscous effect there was a phase difference δ between the applied stress and the measured strain:

$$\sigma \left(t\right)=\sigma_{0}\sin\left(\omega t\right);\varepsilon \left(t\right)=\varepsilon_{0}\sin\left(\omega t-\delta \right)$$

The elastic ${\text{(E}}^{'})$ and viscous ${\text{(E}}^{\text{''}})$ moduli can be calculated as:

$${\text{E}}^{'}=\frac{\sigma_{0}}{\varepsilon_{0}}\text{cos}\left(\delta \right);\;{\text{E}}^{\text{''}}=\frac{\sigma_{0}}{\varepsilon_{0}}\text{sin}\left(\delta \right)$$

When presented as a function of each other, σ(ε)  or  ε(σ), a hysteretic loop will form. If the material is purely elastic, the curve will be linear. If a non-zero viscous element exists, the loop will deviate from a line and have a non-zero width.

To quantify the orientation correlation between the applied stress tensor and the measured growth/strain tensor, we defined θ(r) as the angle between the local principal direction of the applied stress (calculated numerically for each experiment, see Supplementary data: Figure S5 for example), and the measured local principal strain direction. In a well-aligned stress-strain situation, where the measured strain tensor follows the calculated stress tensor, |cos(θ)| , defined here as the stress-strain alignment, is close to 1. When the strain is not correlated with the stress tensor, we expect a mean value of $\left|\cos\left(\theta \right)\right|=\frac{2}{\pi }\approx 0.63,$ the average value of |cos(θ)| if θ is distributed randomly.

## Results and discussion

We first analysed the growth rates under constant load. In these measurements the leaf was kept under constant tensile stress for 24 hours. The applied force was up to 40 mN. We compensated for relaxation as a result of creep or growth.

**Fig. 2.**
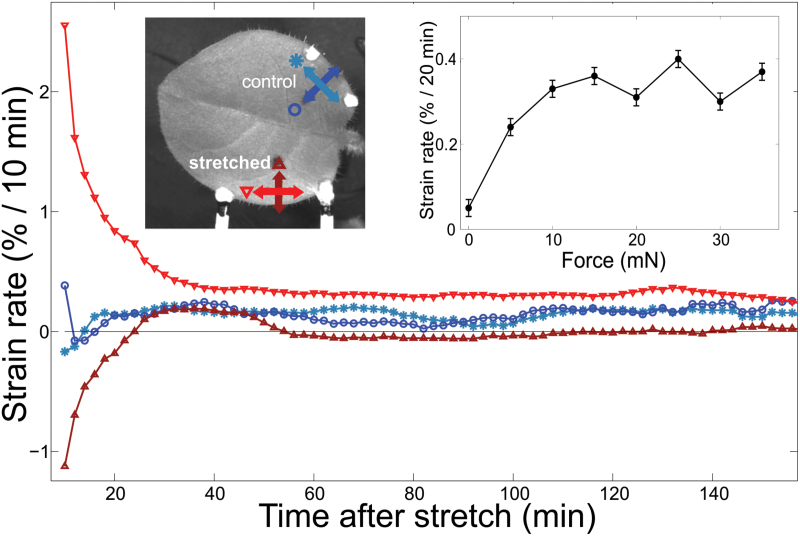
Average growth rates along selected directions in a typical leaf stretched by 40 mN: shown are the longitudinal growth rate within the stretched side, parallel to the principal stress, and leaf edge (red down-pointing triangles) and in the perpendicular direction (brown up-pointing triangles), together with growth rates within the control (unstretched) side (parallel to the leaf edge in blue circles; perpendicular to the leaf edge in light blue stars). Large deformation rates are measured during the first 30 minutes of stretching. These are followed by relatively steady growth rates over time. At all times the growth along the stretched direction is larger than the natural growth rates (on the control side), while the growth rate along the perpendicular direction is smaller. Measurements were taken every 2 minutes. Each point is obtained by averaging the local growth over ~100 points in the relevant ROI, with smoothing in time over 15 sequential frames. **Inset**: Average growth rate within the ROI (excluding the first 40 minutes) in the stretched direction vs. the applied force, measured on a single leaf. Above some threshold (~10 mN in this case) the growth saturates at a maximal value of 0.3% per 20 minutes, even when the load is increased by a factor of 3. These are mean values measured 1–3 hours after the application of stress. Natural changes in the growth rate of the entire leaf are compensated for, based on the growth rate of the control side. The measurement was not performed on the same leaf as in the main figure. Error bars represent the accuracy in determining the average value.

Application of mechanical stress caused high strain rates and initial rapid deformation, which decayed exponentially with a time constant of the order of several minutes (Fig. 2). The tissue elongated in the stretching direction (red down-pointing triangles) by up to 5% over the first 30 minutes. During this time, the tissue hardly grew and even slightly shrank along the perpendicular direction (brown up-pointing triangles). The strain fields were highly correlated with the applied stress, with an average alignment of |cos(θ)|>0.9 [averaged over the region of interest (ROI) seen in Fig 1A]. Unloading the leaf within this period shows that this deformation was mostly of linear viscoelastic nature (see Fig. 4 and DMA measurements below), and demonstrated retarded recovery.

The perpendicular direction, which at first responded by contracting, regained a positive strain rate about 20 minutes after the initiation of loading, and then reached a steady state of nearly-zero strain rate.

For several hours after stretching, a rather constant but anisotropic growth rate was observed. During this time we observed a high stress-strain alignment with |cos(θ)|≈0.85. The relative longitudinal growth rate in the stretch direction (see example in Fig. 2, and more examples in Supplementary data: Figure S7) was on average 1.5%/h, considerably higher than the growth rate in the control side, which was 0.75%/h. Given that the growth rate along the perpendicular direction was nearly zero (~0.1%/h), the total lateral growth rate in the stretched side was only 10% higher than the control side.

Increasing the force applied on the tissue led to an increase in the elongation rate. However, the steady growth rate along the stretched direction saturated and did not increase beyond a maximal rate, even when the applied stress continued to increase (inset in Fig. 2). Because most viscoelastic materials extend faster if the stress exerted on them increases, we believe this saturation was more likely due to biological limitations, such as the rate of degradation and deposition of new material in the cell wall, rather than a pure mechanical process.

The active nature of the growing tissue became evident at longer times, of 12 to 24 hours. When observed at t>12 hours, the alignment decreased to |cos(θ)|≈0.75 and below, approaching the value for isotropic growth (0.63). This indicates that the growth became more isotropic, even though the leaf was subjected to the same force as before. No significant change in the total growth rate was observed. The control side grew isotropically whenever measured (see Supplementary data: Figure S6).

**Fig. 3.**
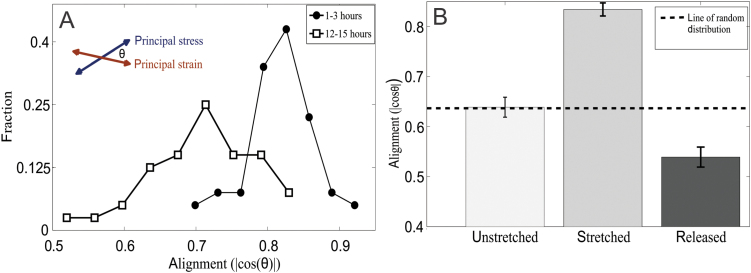
**(A)** Distribution of average stress-strain alignment (see definition in main text) in the stretched area for 50 experiments in two time regions: during the first 3 hours (full circles) and after 12–15 hours (empty squares). The alignment decreases over time when the leaf is subjected to constant tensile stress. **(B)** Average alignment in a freely growing leaf (left), in a leaf stretched by a force of 40 mN for 3–10 hours (middle), and in a leaf released from an overnight stretch of 40 mN (right). These measurements include only steady-state growth rates; the first 45 minutes after stretch or release, during which the initial high strain rates take place, are excluded.

When the stress was released after being held constant for over 15 hours, the leaf growth changed to an opposite anisotropy, that is, growth in the direction that was previously stretched was slower than in the perpendicular direction. This was indicated by the alignment between the previously applied stress and the growth field measured after unloading, which was close to |cos(θ)|≈0.55, and below the random value (Fig. 3B). The leaf recovered its growth isotropy only after several hours, therefore partially compensating for the deformation caused by the long stretching.

**Fig. 4.**
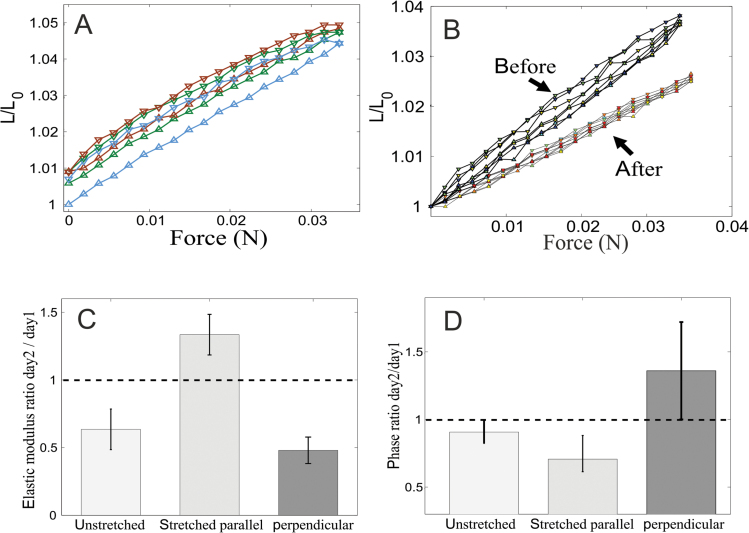
**(A)** An example of raw DMA results. Three consecutive DMA cycles on the same leaf are shown. Each measurement was obtained over relatively short time cycles of 5 minutes so that the natural growth is small compared to the strain amplitude. In the first cycle of loading (blue) we find a significant irreversible deformation (the loop is not closed). No such deformation is observed in the following cycles (green, brown), where the leaf responds viscoelastically to the varying load, and the deformation is linear and reversible. To analyse the viscoelastic parameters, several cycles are performed for each data point, and any mean net deformation is removed from the plot, as seen in B. **(B)** An example of the change in DMA results as a result of stretching (not the same data set as in A). The two groups of consecutive DMA cycles were obtained from the same leaf on two consecutive days, before and after applying constant stretch for 24 hours. The two sets of loops were taken at the same time of the day. A typical decrease in the slope (increasing elastic modulus) after stretching, as well as a decrease in the loop area (decreasing phase lag) can be seen. **(C)** Changes in the elastic modulus due to extended loading measured on 75 plants (25 for each measurement): the elastic modulus E’ normalized by E’ measured 24 hours before, for an unstretched (control, left) leaf, and for a leaf subjected to extended tensile stress. The extended constant load was applied parallel (middle) or perpendicular (right) to the loading in the DMA measurement. The leaf stiffened along the direction of the static stretching and softened in the perpendicular direction. **(D)** Changes in the phase due to extended loading: the phase difference between stress and strain, indicating the inelastic properties of the tissue, measured 24 hours apart. A very small decrease occurs in the control (unstretched) leaf. In the stretched direction a decrease is observed, indicating that the leaf material flows less. In the perpendicular direction the leaf becomes more pliable. Measured on the same plants as in C.

In order to reveal the origin of the observed changes in growth patterns, we performed DMA of the growing tissue, before and after the static loading. The leaf tissue responded viscoelastically to the cyclic loading, with a clear hysteretic loop in the force-strain plane (Fig. 4A, B). Following a first DMA measurement, the leaf was held under a constant tensile force of 40 mN for 23.5 hours. It was then released shortly before the second measurement, so that the two DMA measurements were performed at the same time of the day. As a control, we used leaves that were not subjected to stress between the two DMA measurements. Based on the temporal sinusoidal profiles of the strain and force, we obtained and tracked changes in the elastic modulus *E’* and the non-elastic phase difference tan(δ ) both in the direction of the stretching and perpendicular to it. Each measurement was performed on 25 leaves, and the average values of seven consecutive cycles (5 minutes each) were used for each data point.

The ratio between the elastic modulus after and prior to the static loading (Fig. 4C) was used to determine the change occurring overnight, where $\frac{E'(day\;2)}{E'(day\;1)}<1$ indicates that the leaf has become more pliable, and $\frac{E'(day\;2)}{E'(day\;1)}>1$ indicates that the leaf has stiffened. We found that young leaves which were not stretched exhibited a natural decrease in their elastic modulus from day to day, with a mean ratio of 0.6. However, when tensile stress was applied, the elastic modulus along the direction of stretching increased considerably. A mean ratio of 1.3 indicated stiffening of the leaf tissue along the direction of static stress. In contrast, when measuring the elastic modulus perpendicular to the direction of the applied stress, we observed a decrease in the elastic modulus, with a mean ratio of 0.5.

When examining the inelastic behaviour of the leaf (Fig. 4D) we measured the phase difference $tan\left(\delta \;\right)=\frac{E''}{E'}$ (typical values of δ are ~0.1 radian) before and after application of the extended load. We observed a small, almost negligible, overnight decrease in δ of the control (unstretched) leaf with a ratio of 0.9±0.1. Prolonged stretching resulted in a more significant decrease in the stretched direction, and an increase in the perpendicular direction. This implies that the leaf not only becomes elastically stiffer (Fig. 4C), but also flows less in the stretched direction, so that any deformation can be easily reversed. However, in the perpendicular direction it became more pliable.

These measurements provide direct evidence that prolonged mechanical stress leads to modification of the leaf’s mechanical properties, making them anisotropic. These changes in the leaf mechanics may explain the changes in the rate and orientation of growth that are presented in Fig. 2, 3.

In conclusion, we can say that a leaf responds to mechanical stress by locally altering its mechanical properties, that is, its elastic and loss modules. These changes affect the rate and directionality of the tissue growth. The changes are nonlinear and involve different timescales. At short times, the tissue yields, releasing stress via rapid deformation, as a soft passive material would do. At longer times, on a scale of several hours, a partial correlation is maintained between the local growth field and the stress field. However, a strong nonlinearity in the leaf response is seen, showing a saturation of the growth rate when the applied stress increases. If the load persists for several hours, a change is observed in the leaf’s mechanical properties; it stiffens along the principal loading direction and softens perpendicularly. As a result of this tissue remodelling, the growth gradually becomes more isotropic, despite the continuous anisotropic external stress. The effect of these modifications in the tissue’s properties persists for several hours after loading has ceased. The combination of changes of different timescales could allow the leaf to regulate its growth, while avoiding the accumulation of large irregular local stresses. Rapid yielding is necessary for local stress release. However, if such local yielding persists for a long time the leaf will be deformed and will not attain its normal shape. Therefore, reinforcing the strained tissue over a long time prevents excess local deformation.

Our measurements show that the leaf is an active material, equipped with mechanisms which allow it to reach its normal shape. The complexity of the living tissue can be approximated to some level as local changes in growth dynamics and material properties that are directly related to the mechanical stimulation the leaf is subjected to.

The results presented in this work show typical changes in growth rate and orientation, as well as in the mechanical properties, with characteristic timescales, in response to external loading. This could serve as a basis for the formulation of equations of motion to describe the dynamics of the growth field.

Our findings may be relevant to the auxin-regulated mechanism, involving also the activity of microtubules and resulting in re-orientation of cellulose fibrils, as described in Sampathkumar *et al.* (2014). We assume that changes in the orientation of cellulose microfibers (Suslov and Verbelen, 2006; Corson *et al.*, 2009) as well as in the cell wall matrix as a whole, and in particular in pectin (Rounds *et al.*, 2011; Palin and Geitmann, 2012), can be expected. The variety of timescales involved leads us to expect multiple biological and physical processes.

These results indicate the need for further study to determine the biological processes underlying the observed dynamic and mechanical properties, in a way that will lead to deeper understanding of the fascinating process of growth regulation.

## Supplementary data

Further details of the experimental setup can be viewed at *JXB* online.

Figure S1. Experimental setup for three-dimensional growth measurements.

Figure S2. Calibration of the applied force.

Figure S3. Example of three-dimensional visualization.

Figure S4. Example of typical measured growth field of a stretched field over the first 20 minutes.

Figure S5. Example of typical calculated stress field.

Figure S6. The alignment of anisotropic growth vs. isotropic growth with the calculated stress field.

Figure S7. Additional growth rate curves.

Supplementary Data

## References

[CIT0001] Alberts B Johnson A Lewis J Raff M Roberts K Walter P 2002 The plant cell wall. New York: Garland Science http://www.ncbi.nlm.nih.gov/books/NBK26928/.

[CIT0002] Armon S Efrati E Kupferman R Sharon E 2011 Geometry and mechanics in the opening of chiral seed pods. Science 333, 1726–1730. 21940888 10.1126/science.1203874

[CIT0003] Bozorg B Krupinski P Jönsson H 2014 Stress and strain provide positional and directional cues in development. PLoS Computational Biology 10, e1003410. 24415926 10.1371/journal.pcbi.1003410 PMC3886884

[CIT0004] Braam J 2005 In touch: plant responses to mechanical stimuli. The New Phytologist 165, 373–389. 15720650 10.1111/j.1469-8137.2004.01263.x

[CIT0005] Cohen J 1995 Lisianthus leaf curl a new disease of Lisianthus caused by tomato yellow leaf curl virus. Plant Disease 79, 416.

[CIT0006] Corson F Hamant O Bohn S Traas J Boudaoud A Couder Y 2009 Turning a plant tissue into a living cell froth through isotropic growth. Proceedings of the National Academy of Sciences 106, 8453–8458. 10.1073/pnas.0812493106 PMC2688973 19423667

[CIT0007] Cosgrove DJ 1993 How do plant cell walls extend? Plant Physiology 102, 1–6. 11536544 10.1104/pp.102.1.1 PMC158739

[CIT0008] Cosgrove DJ 2005 Growth of the plant cell wall. Nature Reviews. Molecular Cell Biology 6, 850–861. 16261190 10.1038/nrm1746

[CIT0009] Coutand C Julien JL Moulia B Mauget JC Guitard D 2000 biomechanical study of the effect of a controlled bending on tomato stem elongation: global mechanical analysis. Journal of Experimental Botany 51, 1813–1824. 11113160 10.1093/jexbot/51.352.1813

[CIT0010] Dumais J Forterre Y 2012 ‘Vegetable dynamicks’: the role of water in plant movements. Annual Review of Fluid Mechanics 44, 453–478.

[CIT0011] Efrati E Sharon E Kupferman R 2009a. Elastic theory of unconstrained non-euclidean plates. Journal of the Mechanics and Physics of Solids 57, 762–775.

[CIT0012] Efrati E Sharon E Kupferman R 2009b. Buckling transition and boundary layer in non-euclidean plates. Physical Review E 80, 016602. 10.1103/PhysRevE.80.016602 19658827

[CIT0013] Elbaum R Zaltzman L Burgert I Fratzl P 2007 The Role of wheat awns in the seed dispersal unit. Science 316, 884–886. 17495170 10.1126/science.1140097

[CIT0014] Elsner J Michalski M Kwiatkowska D 2012 Spatiotemporal variation of leaf epidermal cell growth: a quantitative analysis of *Arabidopsis thaliana* wild-type and triple cyclind3 mutant plants. Annals of Botany 109, 897–910. 22307569 10.1093/aob/mcs005 PMC3310487

[CIT0015] Forterre Y Skotheim JM Dumais J Mahadevan L 2005 How the Venus flytrap snaps. Nature 433, 421–425. 15674293 10.1038/nature03185

[CIT0016] Gibson WT Gibson MC 2012 Growing cells push back under pressure. Cell 149, 259–261. 22500795 10.1016/j.cell.2012.03.019

[CIT0017] Gjorevski N Nelson CM 2010 The mechanics of development: models and methods for tissue morphogenesis. Birth Defects Research. Part C, Embryo Today: Reviews 90, 193–202. 10.1002/bdrc.20185 PMC3087175 20860059

[CIT0018] Hellgren JM Olofsson K Sundberg B 2004 Patterns of auxin distribution during gravitational induction of reaction wood in poplar and pine. Plant Physiology 135, 212–220. 15122024 10.1104/pp.104.038927 PMC429355

[CIT0019] Jaffe MJ 1980 Morphogenetic responses of plants to mechanical stimuli or stress. BioScience 30, 239–243.

[CIT0020] Kierzkowski D Nakayama N Routier-Kierzkowska A-L Weber A Bayer E Schorderet M Reinhardt D Kuhlemeier C Smith RS 2012 Elastic domains regulate growth and organogenesis in the plant shoot apical meristem. Science 335, 1096–1099. 22383847 10.1126/science.1213100

[CIT0021] Mirabet V Das P Boudaoud A Hamant O 2011 The role of mechanical forces in plant morphogenesis. Annual Review of Plant Biology 62, 365–385. 10.1146/annurev-arplant-042110-103852 21332360

[CIT0022] Nakayama N Smith RS Mandel T Robinson S Kimura S Boudaoud A Kuhlemeier C 2012 Mechanical regulation of auxin-mediated growth. Current Biology 22, 1468–1476. 22818916 10.1016/j.cub.2012.06.050

[CIT0023] Nath U Crawford BCW Carpenter R Coen E 2003 Genetic control of surface curvature. Science 299, 1404–1407. 12610308 10.1126/science.1079354

[CIT0024] Onoda Y Schieving F Anten NPR 2015 A novel method of measuring leaf epidermis and mesophyll stiffness shows the ubiquitous nature of the sandwich structure of leaf laminas in broad-leaved angiosperm species. Journal of Experimental Botany 66, 2487–2499. 25675956 10.1093/jxb/erv024 PMC4986859

[CIT0025] Palatnik JF Allen E Wu X Schommer C Schwab R Carrington JC Weigel D 2003 Control of leaf morphogenesis by microRNAs. Nature 425, 257–263. 12931144 10.1038/nature01958

[CIT0026] Palin R Geitmann A 2012 The role of pectin in plant morphogenesis. Bio Systems 109, 397–402. 10.1016/j.biosystems.2012.04.006 22554809

[CIT0027] Paredez AR Somerville CR Ehrhardt DW 2006 Visualization of cellulose synthase demonstrates functional association with microtubules. Science 312, 1491–1495. 16627697 10.1126/science.1126551

[CIT0028] Poethig RS Sussex IM 1985 The developmental morphology and growth dynamics of the tobacco leaf. Planta 165, 158–169. 24241040 10.1007/BF00395038

[CIT0029] Rounds CM Lubeck E Hepler PK Winship LJ 2011 Propidium iodide competes with Ca(2+) to label pectin in pollen tubes and Arabidopsis root hairs. Plant Physiology 157, 175–187. 21768649 10.1104/pp.111.182196 PMC3165868

[CIT0030] Sampathkumar A Yan An Krupinski P Meyerowitz EMM 2014 Physical forces regulate plant development and morphogenesis. Current Biology 24, R475–R483. 24845680 10.1016/j.cub.2014.03.014 PMC4049271

[CIT0031] Sharon E Efrati E 2010 The mechanics of non-euclidean plates. Soft Matter 6, 5693.

[CIT0032] Shawky JH Davidson LA 2015 Tissue mechanics and adhesion during embryo development. Developmental Biology 401, 152–164. 25512299 10.1016/j.ydbio.2014.12.005 PMC4402132

[CIT0033] Suslov D Verbelen J-P 2006 Cellulose orientation determines mechanical anisotropy in onion epidermis cell walls. Journal of Experimental Botany 57, 2183–2192. 16720609 10.1093/jxb/erj177

[CIT0034] Uyttewaal M Burian A Alim K Landrein B Borowska-Wykręt D Dedieu A 2012 Mechanical stress acts via katanin to amplify differences in growth rate between adjacent cells in Arabidopsis. Cell 149, 439–451. 22500806 10.1016/j.cell.2012.02.048

[CIT0035] Wolff J 2012 The Law of Bone Remodelling. Springer Science & Business Media https://books.google.com/books?hl=en&lr=&id=nYdfBgAAQBAJ&pgis=1.

