# Supplementary material for: The rheology of a growing leaf: stress-induced changes in the mechanical properties of leaves
Source: J Exp Bot. 2016 Sep 20;67(18):5509–15. doi: 10.1093/jxb/erw316 (PMC5049397; doi:10.1093/jxb/erw316)
Supplement: Supplementary Data [file supp_67_18_5509__index.html]

The rheology of a growing leaf: stress-induced changes in the mechanical properties of leaves — The rheology of a growing leaf: stress-induced changes in the mechanical properties of leaves — Supplementary Data 

# The rheology of a growing leaf: stress-induced changes in the mechanical properties of leaves

## Supplementary Data

Data files

- Supplementary\_Figures\_S1\_S7.pdf - Supplementary Data
