# Supplementary material for: The rheology of a growing leaf: stress-induced changes in the mechanical properties of leaves
Source: J Exp Bot. 2016 Sep 20;67(18):5509–15. doi: 10.1093/jxb/erw316 (PMC5049397; doi:10.1093/jxb/erw316)
Supplement: Supplementary Data [file supp_erw316_Supplementary_Figures_S1_S7.pdf]

Supplementary material for " The Rheology of a Growing Leaf"

List of figures:

S1: experimental setup for 3D growth measurements.

S2: calibration of the applied force

S3 example of 3D visualization,

S4: example of typical measured growth field of a stretched field over the first 20 minutes

S5: example of typical calculated stress field

S6: the alignment of anisotropic growth vs. isotropic growth with the calculated stress field.

S7: additional growth rate curves

Methods and Experimental Setup

To study the effect of mechanical stress, an experimental setup was built which allows the application of controlled tensile stress to a leaf, while gathering information on the surface and 3D configuration of the leaf.

General setup description (fig. S1): The setup consists of a stress-control system, and two cameras which collect data for growth analysis. The images are then processed using PIV (particle image velocimetry) technique to measure the growth (strain tensor) with temporal resolution on the order of minutes, and spatial resolution on the order of tens of microns. The applied stress field for each experiment is calculated numerically with finite element method to provide local, as well as global, stress-strain relations. All measurement described in this work were performed on young growing leaves of 1-month old plants of *Nicotiana Tabacum* while still intact and attached to the plant.

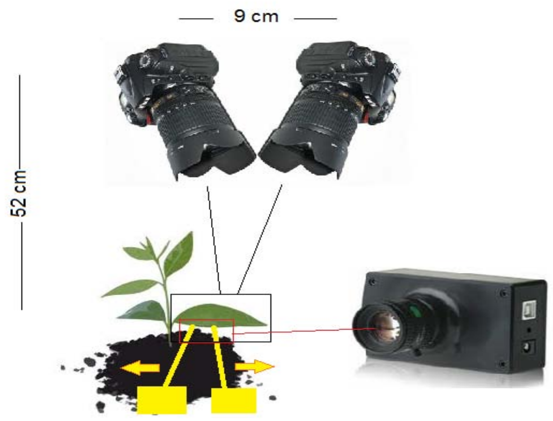

Fig.S1. Experimental setup**:** elastic glass tubules (in yellow) mounted on motorized stages moving at opposite directions are used to stretch one side of the leaf. Cameras monitor the applied force (red square) and acquire images for data acquisition (black square).

Stress control: the leaf is glued at its edge to two flexible glass tubules held by micromanipulators mounted on a motorized stage, and then stretched between the two. The tension causes a deflection of the tubules, which serves as an indicator of the applied force. We used Sutter Instruments borosilicate glass tubules with an outer diameter of 1 mm and inner diameter of 0.78-0.5 mm (thicker tubules apply more force for a given displacement). The tubules were 6 cm long. To calibrate the force applied by them, one end of the tubule was controlled manually by a micromanipulator while the other end was pressing on a sensitive scale. A calibration curve can be seen in fig. S2. Two-part 5-minutes Super Glue epoxy was used to attach the tubules to the edge of the leaf. This glue was chosen since upon dry down it is much more rigid than the leaf tissue and so does not affect the measurement of mechanical properties. It also showed no evidence of damage to the leaf, and seemed to create a clamp around the tissue, attaching mostly to the trichomes.

The position of the tubules' tips is monitored by a Lumenera LW575c camera, and is re-positioned to compensate for stress relaxation due to leaf growth or creep, thus maintaining the required stress throughout the measurement. The force used for the experiments described in this work is of the order of 40 millinewton. Once the force is set, it can be kept constant or varied with a sinusoidal profile, with a resolution of about 0.1 millinewton. This is determined by the resolution of the image on the camera.

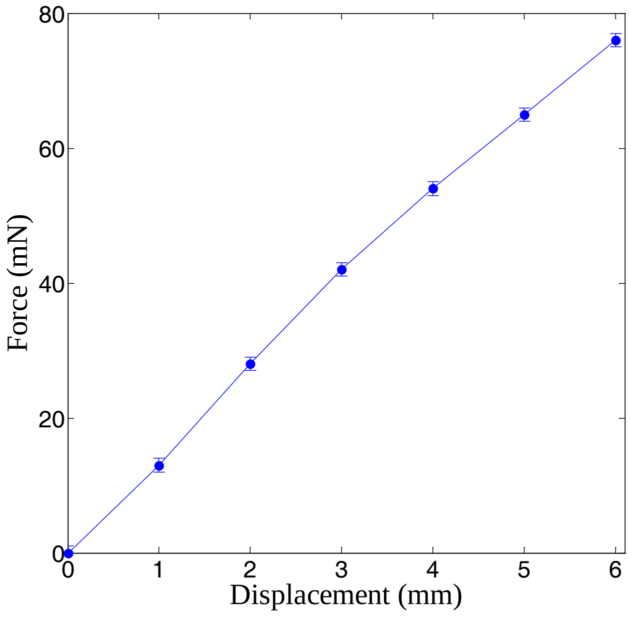

Fig S2: A calibration curve for the force applied by the deflection of the end of a 6 cm-ling glass tubule with outer diameter of 1 mm and inner diameter of 0.78 mm.

3D and Growth Analysis:

Collecting data from the free side is challenging, since while the stretched side is straight and stays put, the free can move. Thus, 3D information is required for full analysis of both sides. To acquire full growth information on the whole leaf, two Lumenera LW575m cameras, with 5.5 mega pixels and 60 mm Micro Nikkor lenses are mounted 52 cm above the leaf. The two cameras are separated by 9 cm so they are oriented with ~10 degrees angle difference.. We track the motion of natural features on the leaf surface, such as veins and trichomes (hairs), to calculate z(x,y) (fig. S3) and the growth tensor (fig.S4). We use PIV (particle image velocimetry) technique, which was mentioned briefly in the main text. This technique searches for the best cross-correlation between images, starting from the entire image and increasing the resolution gradually. It provides the displacement field, which indicates where each point in one image has moved to in the other image. This can be used both for the Z(x,y) calculation (more details follow) and for calculating the growth (strain) tensor.

The growth field is then compared to the stress tensor which is calculated numerically (fig S5). The calculated z(x,y) is used both for calculating the metric of the leaf, and to compensate for the apparent growth resulting from changes in the distance between the leaf and the camera, which happens due to movements that occur during growth. Z resolution is approximately 80 microns.

Z(x,y) calculation

To obtain the surface of the leaf, two images taken at the same time by the two cameras are compared using PIV, so an apparent displacement field is obtained. This field gives the difference in the position of each point in the images, resulting from the difference in the angle of view. This apparent displacement can be traced back to the Z position of each point using simple geometrical calculation of the parallax. Having achieved a Z position of each point, bad points are removed and a 2D polynomial surface of the best fit is found. This smoother surface is used as the basis for calculating the leaf metric so that the growth can be obtained.

Correcting apparent growth: a correction is applied for apparent growth resulting from movement of the plants that lead to a change in the distance between the cameras and the leaf. As a result of such movement, features in subsequent images can appear to change their size and lead to false apparent growth. The apparent growth can be considered approximately linear in the range of plant movement (~1 cm). A calibration of this apparent growth in the relevant spatial scale was performed, and the correction is applied locally according to the change in z(x,y) of every data point at any given time interval t_2- t_1.

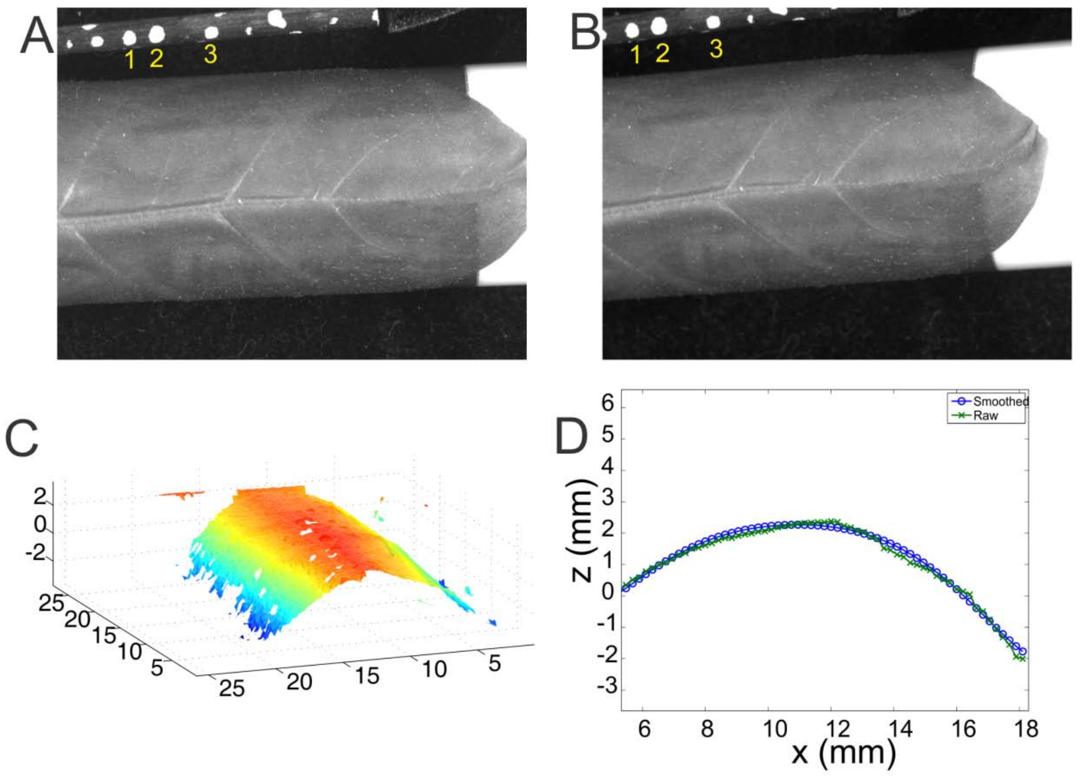

Fig.S3 : A Stereoscopic measurement. A Tobacco leaf is tightly rolled on a cylinder of radius 8.5 mm for demonstration of measurement technique. A), B) – The two images obtained by the cameras. Three white dots on a stick, which determines the $z = 0$ plane are marked in both images. C) The raw 3D surface obtained from stereoscopic analysis of the two images (scales in mm). D) The raw surface profile along a line (green crosses) and the smooth curve obtained from polynomial fit of the surface (blue circles).

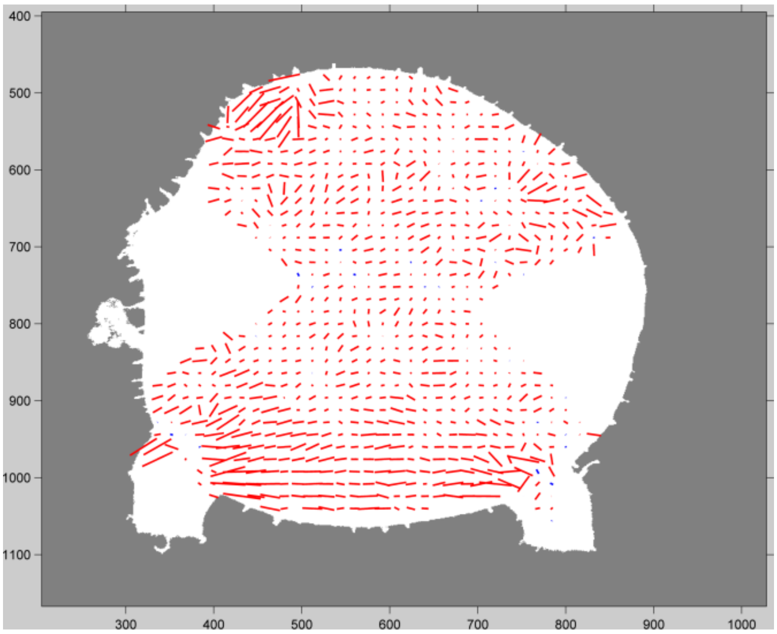

Fig S4: The principal local strain (growth) on the same loaded leaf. High level of alignment appears in regions of high stress, while the orientation of growth is more random in other regions. This measurement shows the growth during the first 20 minutes after tensile stress is applied.

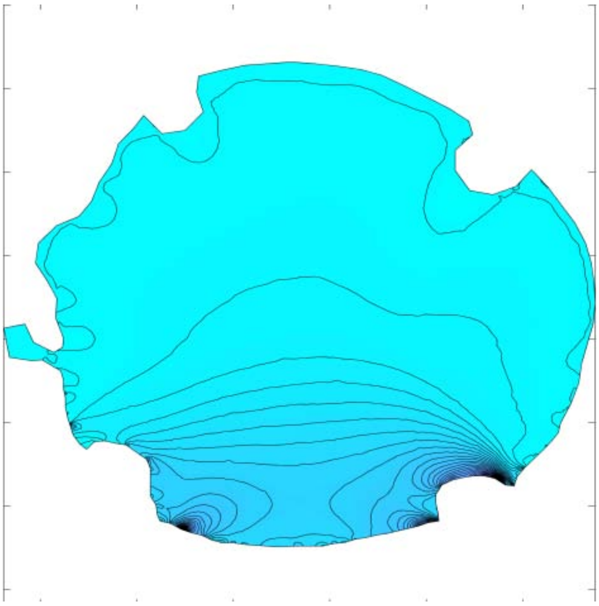

Fig S5: Calculation of the stress on the loaded leaf. Color indicates the magnitude of the large principal stress and lines indicate equal stress levels.

Growth calculation: images taken at different times t_1, t_2 are compared and PIV is used to calculate the displacement field, showing the direction and magnitude of the changes between subsequent images, . These changes represent any growth or deformation that occur during this time interval. The metric, g_1 at time t_1 is calculated locally for each data point, based on x,y and ,z(x,y). We find O and K , which normalize and diagonalize g_1, so that:

$$O^T g_1 O = \begin{pmatrix} \lambda_1 & 0 \\ 0 & \lambda_2 \end{pmatrix}; \ K = \begin{pmatrix} \sqrt{\frac{1}{\lambda_1}} & 0 \\ 0 & \sqrt{\frac{1}{\lambda_2}} \end{pmatrix}; \ O^T K^T g_1 K O = I$$

Then the metric $g_{2,}$ at a later time $t_{2,}$ is calculated at the point to which the point has moved according to the displacement calculated by the PIV. For that purpose, z is interpolated to this new position. The change $\frac{g_2}{g_1}$ is defined as the growth tensor. To calculates it, g_2 is written in the above basis in which g_1 is written as I, and the relative change is then the growth tensor G. We find U which diagonalizes G so that it can be written in the form:

$$G = U^T O^T K^T g_2 K O U = \begin{pmatrix} \tilde{\lambda}_1 & 0 \\ 0 & \tilde{\lambda}_2 \end{pmatrix}$$

Stress calculation: The stress field of the entire leaf is calculated using finite elements method with Matlab’s PDE (partial differential equations) tool. This is don based on the image taken by the camera and assuming free ends through the leaf, except the points held by the tubules in which a known force is applied. Certain assumption need to be made regarding the properties of the leaf, in order for the stress to be accurate in magnitude, however, we have no interest in the absolute magnitude of the stress but rather in its spatial distribution and the relative magnitude of its tensor components.

An example of a stress field is seen in fig S5 .

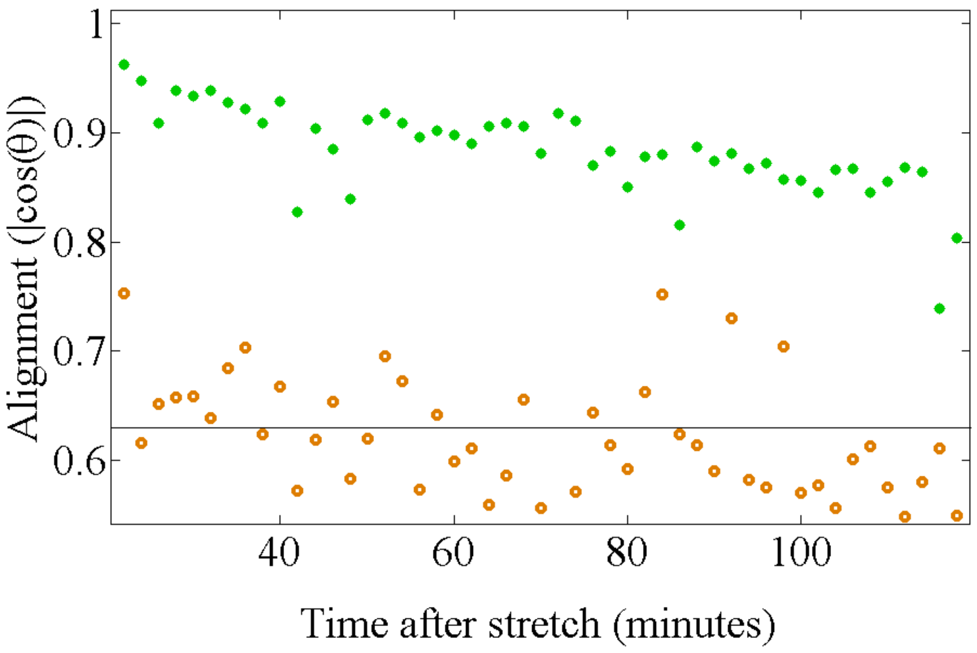

Fig. S6: The alignment between the calculated stress field and the measured strain field of a stretched leaf (green full circles) and unstretched leaf (orange empty circles). Both strain fields were compared to the same stress pattern. When the leaf is not stretched (i.e. the stress is not applied) the strain is random and isotropic, and therefore uncorrelated with the stress. In this case, the alignment is expected to be distributed around $|\cos(\theta)| = \frac{2}{\pi} \approx 0.63$, as is seen here (black solid line). When the leaf is stretched, the growth become unisotropic and aligned with the stress field, resulting in a value of $|\cos(\theta)| > \frac{2}{\pi}$.

Noise and limitations

Our method is based on tracking minor changes in the leaf surface. Although in principle it is possible to take measurement that are separated by very short times, in practice, our ability to detect changes is limited by the resolution of the image. Sub-pixel changes cannot be tracked accurately and create local noise, which appears as abrupt local zero-sum changes in growth. However, when averaging over an area, mean values with high accuracy can be obtained. A calibration measurement over dead and dried leaf tissue yielded a zero longitudinal growth rate with accuracy higher than 0.01% per 20 minutes.

In fig. S7 several examples of typical results are seen, similar to the one shown in fig 2 in the main text. Note that each point is averaged over an area as shown in fig. 1 of the main text

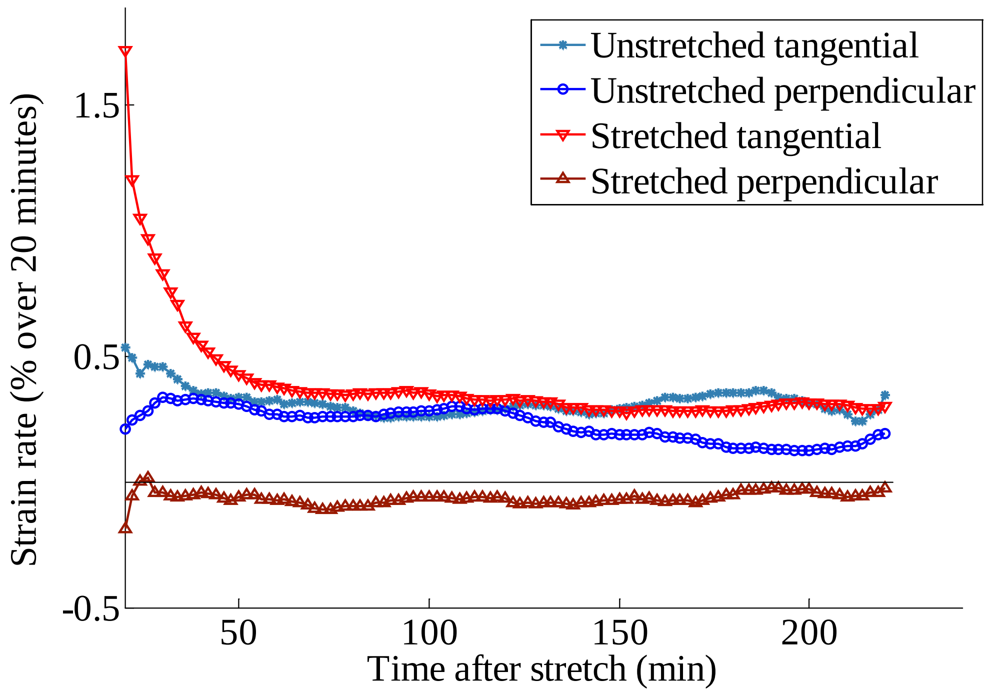
Unstretched tangential
Unstretched perpendicular
Stretched tangential
Stretched perpendicular
Strain rate (% over 20 minutes)
1.5
0.5
-0.5
50
100
150
200
Time after stretch (min)

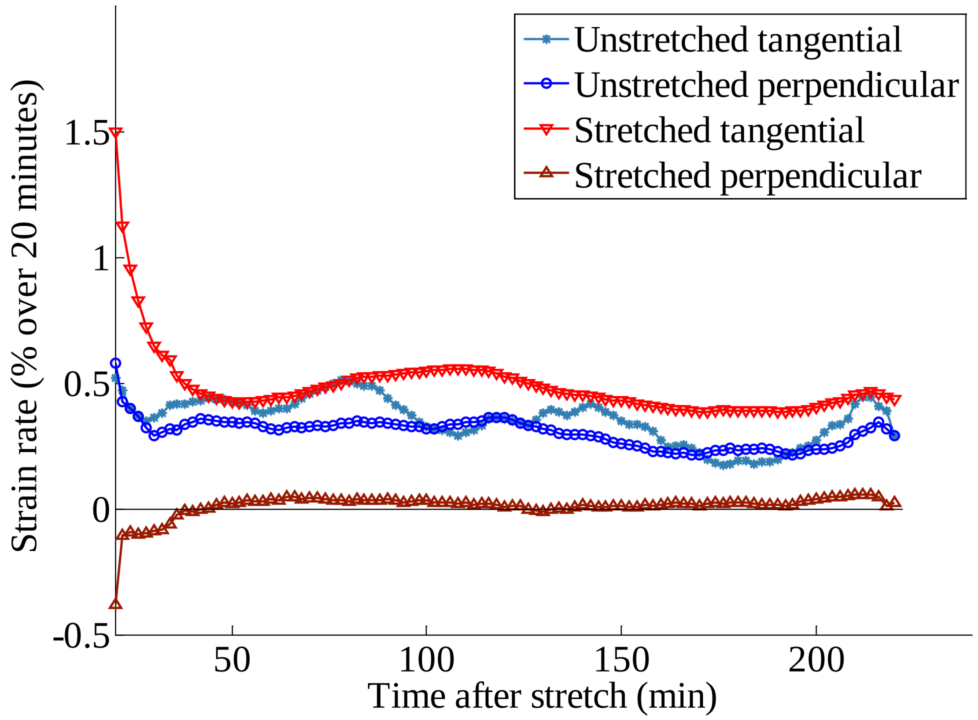
Unstretched tangential
Unstretched perpendicular
Stretched tangential
Stretched perpendicular
Strain rate (% over 20 minutes)
1.5
1
0.5
0
-0.5
50
100
150
200
Time after stretch (min)

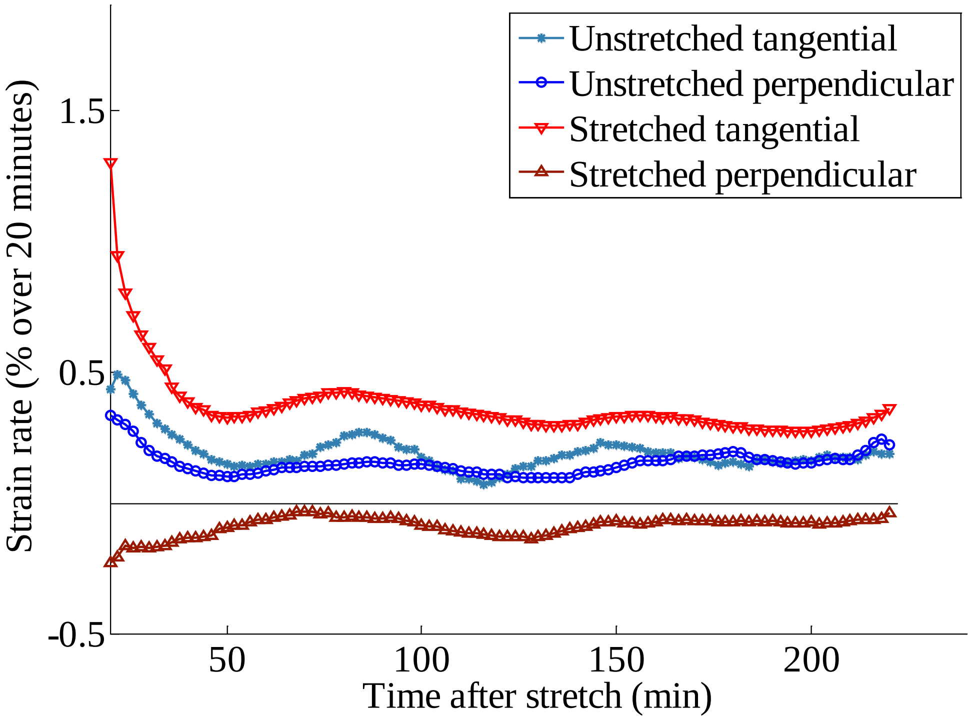

Fig S7: examples of strain rates of several leaves, for a few hours after tensile stress is applied.

DMA measurements

For the measurement of mechanical properties, the two glue points which attach the tubules to the leaf are marked with bright color so that force and strain can be tracked in real time. A Sinusoidal force profile is applied. The force is measured by moving one tubule while observing the deflection of the other. As explained above, the deflection of the elastic tubule is linearly related to the force, and so can serve as a force gauge. The strain is measured by tracking the distance between the two glue points. In this way, $F(t)$ *and* $\varepsilon(t)$ are obtained, and $\varepsilon(F)$ is calculated, which is shown in fig. 4 of the main text.

The accuracy in the measurement is determined by the resolution of the camera, and, as mentioned before, is of the order of 0.1 millinewton.
